# Similarities between bacterial GAD and human GAD65: Implications in gut mediated autoimmune type 1 diabetes

**DOI:** 10.1371/journal.pone.0261103

**Published:** 2022-02-23

**Authors:** Suhana Bedi, Tiffany M. Richardson, Baofeng Jia, Hadeel Saab, Fiona S. L. Brinkman, Monica Westley

**Affiliations:** 1 Department of Natural Sciences and Mathematics, The University of Texas at Dallas, Richardson, TX, United States of America; 2 Department of Molecular Physiology and Biophysics, Vanderbilt University, Nashville, TN, United States of America; 3 Department of Molecular Biology and Biochemistry, Simon Fraser University, Burnaby, CA, United States of America; 4 Intern, The(sugar)science, Los Angeles, CA, United States of America; 5 Founder, The(sugar)science, Los Angeles, CA, United States of America; La Jolla Institute for Allergy and Immunology, UNITED STATES

## Abstract

A variety of islet autoantibodies (AAbs) can predict and possibly dictate eventual type 1 diabetes (T1D) diagnosis. Upwards of 75% of those with T1D are positive for AAbs against glutamic acid decarboxylase (GAD65 or GAD), a producer of gamma-aminobutyric acid (GABA) in human pancreatic beta cells. Interestingly, bacterial populations within the human gut also express GAD and produce GABA. Evidence suggests that dysbiosis of the microbiome may correlate with T1D pathogenesis and physiology. Therefore, autoimmune linkages between the gut microbiome and islets susceptible to autoimmune attack need to be further elucidated. Utilizing *in silico* analyses, we show that 25 GAD sequences from human gut bacterial sources show sequence and motif similarities to human beta cell GAD65. Our motif analyses determined that most gut GAD sequences contain the pyroxical dependent decarboxylase (PDD) domain of human GAD65, which is important for its enzymatic activity. Additionally, we showed overlap with known human GAD65 T cell receptor epitopes, which may implicate the immune destruction of beta cells. Thus, we propose a physiological hypothesis in which changes in the gut microbiome in those with T1D result in a release of bacterial GAD, thus causing miseducation of the host immune system. Due to the notable similarities we found between human and bacterial GAD, these deputized immune cells may then target human beta cells leading to the development of T1D.

## Introduction

Glutamic acid decarboxylase (GAD65) is a prominent autoantibody in type 1 diabetes (T1D). For those diagnosed as a teen, in addition to having an HLA DR3/DR2 signature, it is likely to be the first autoantibody clinically detected in the blood. GAD65 antibodies are prevalent in most T1D cases, occurring in over 70% of patients [1]. Autoantibodies directed against GAD65 recognize linear and conformational epitopes throughout the protein. The functional region of GAD65 contains a catalytic group involving several residues that are distantly located in the primary amino acid sequence of the protein. The enzymatic function of GAD65 and subsequent regulation of GABA production is dependent on a key residue within the catalytic loop, Lys396 [2]. This residue covalently binds pyridoxal 5’-phosphate (PLP), a co-factor essential for GABA production by GAD65. The pyridoxal-dependent decarboxylase (PDD) domain in GAD65 is necessary for the enzymatic generation of GABA. A reduction in PLP levels through deficiencies in its precursor, vitamin B6, may be implicated in GAD65 initiated autoimmunity and subsequent T1D onset [3]. Thus, T1D-related immune recognition of GAD65 is highly dependent on the exposure of these regions to autoreactive antigen-presenting cells (APCs). These activated APCs can then attack GAD65-expressing pancreatic beta cells leading to their destruction and eventual T1D diagnosis.

In addition to beta cells, many bacteria within the gut express GAD (bacterial nomenclature for human GAD65). While there is great diversity within the gut microbiota, more than 95% of gut bacterial species fall into four major microbial phyla: Firmicutes, Bacteroidetes, Actinobacteria, and Protecteobacteria [4]. Bifidobacterium adolescentis, in particular, is an important gut bacterial GABA producer and has the highest prevalence of gad genes in their genomes [5]. These bacterial species provide a homeostatic environment that fosters and facilitates gut epithelial integrity and immunity. Varied literature supports a decrease in microbial diversity and an increase in a “leaky gut” phenotype throughout T1D [6–8]. GABA producing Bifidobacterium bacterial counts drop as the Firmicutes/Bacteroides (F/B) ratios decrease in T1D [9]. There is also a reduction in the abundance of Clostridium clusters IV and XIVa and mucin-degrading bacteria such as Prevotella and Akkermansia. Butyrate is an important metabolite produced by these symbiotic bacterial populations. Butyrate supports gut integrity and has beta cell-specific anti-inflammatory effects [10–12]. Reducing these microbes and their byproducts may contribute to why children at risk for T1D have higher intestinal permeability and T1D-associated gut dysbiosis.

One hypothesis for the interrelationship between dysbiosis and T1D is that as these bacterial populations die, they release GAD65 mimetics that trigger the immune system. Presumably, as GABA-producing bacteria like B. adolescentis die from antibiotic misuse or other pathology, immune cells located in Peyer’s patches and deep in the enterocyte border detect the inappropriate presence of GAD. Thus, APCs can further inform CD8+ T cells in nearby lymph nodes. The lymphatic pathways between the intestinal submucosa and pancreatic lymph nodes may provide a route for these “miseducated” CD8+ T cells to access GAD65+ beta cells. GAD65 may also originate from beta cells leading to an increase in epitope spreading and a misguided immune attack on these insulin-producing cells.

To initiate the exploration of this hypothesis, we utilized *in silico* methodologies to compare gut microbial species that decline at the onset of T1D and express GAD with GAD65 sequences in human pancreatic beta cells. We show notable sequence similarity, including relevant motif and T cell epitope overlaps between several bacterial species and beta cells, particularly in the functionally important pyridoxal dependent decarboxylase (PDD) domain of GAD65. Our *in silico* exercise suggests that further investigation is warranted of the relationship between microbial disappearance and appearance of GAD65 autoantibodies during the prodrome and presentation of T1D.

## Materials and methods

### GAD sequence and T cell receptor epitope curation

Twenty-five bacterial GAD proteins from species of interest, including mice, chimpanzee, and human GAD65 sequences, were curated from the NCBI genes database (Table 1). The bacterial species were chosen by considering their association with the gut microbiome, their significance in the gut microbiome alterations seen in T1D patients, human-related pathogen status, and non-human-related environmental species. GAD65 T cell receptor epitopes were curated from a literature review [13].

**Table 1:** GABA producing bacterial species and their respective GAD gene.

Category	Organism	Gene ID
Human	Homo sapiens	2572
Primate	Pan troglodytes	466026
Mouse	Mus musculus	14417
T1D	Streptococcus pneumoniae	Z49109.1
T1D	Bifidobacterium moukalabense	61040350
T1D	Bifidobacterium adolescentis	56675515
T1D	Bacteroides fragilis	61595025
T1D	Ruminococcus	Genome not found
T1D	Veillonella	Genome not found
T1D	Blautia	Genome not found
T1D	Lachnospira	Genome not found
T1D	Alistipes obesi	Genome not found
T1D	Clostridium spp.	29570578
T1D	Prevotella	Genome not found
T1D	Parabacteroides distasonis	CP040468
Gut	Escherichia coli	946058
Gut	Listeria monocytogenes	985123
Gut	Salmonella enterica	13913147
Gut	Streptomyces sp. NRRL B-24085	6211654
Gut	Carnobacterium maltaromaticum	56848084
Gut	Nocardia brasiliensis	13733698
Gut	Lactobacillaceae	56991876
Pathogen	Gammaproteobacteria	QNEL01000191.1
Pathogen	Klebsiella pneumoniae IS22	12545741
Environment	Planctomycetes bacterium	KAA3606119.1
Environment	Sediminibacterium sp.	JADMJG010000006.1
Environment	Moorea producens	CP017599.1
Environment	Magnetococcales bacterium	JADGBX010000054.1
Environment	Scytonema hofmannii	ANNX02000047.1
Environment	Candidatus Aminicenantes bacterium	WVZL01000014.1
Environment	Bdellovibrio sp. qaytius	CP025734.1
Environment	Verrucomicrobia	NZEQ01000004.1

All GAD sequences in bacteria and the three mammalian species under consideration were obtained by performing a BLASTP search, with human GAD65 as the query sequence. Furthermore, only those sequences with significant e and p values were well annotated on the NCBI database and/or RefSeq and were identified as GAD proteins were considered. All other hits, including unnamed protein products, were ignored.

### Multiple sequence alignment and motif identification

Multiple sequence alignment was performed using ClustalW (Ver. 1.2.2) with a gap opening penalty of 10 and a gap extension penalty of 0.2 [14]. The resulting alignment is visualized and annotated with CLC Sequence Viewer (Ver. 8.0) to highlight conservation and amino acid properties. Identification of ungapped motifs was done using the MEME tool (Ver. 5.4.1) under the MEME suite tools for motif discovery and enrichment [15]. The output consisted of 10 motifs, shared by various (not necessarily all) organisms into consideration. The motifs identified for each organism were then ordered based on the phylogenetic tree constructed for the sequences under consideration, using the Simple Phylogeny Tool [16]. The phylogenetic tree was re-rooted at Homo sapiens using iTol, and the resulting tree was exported locally [17]. Subsequently, each motif was run against protein families, using a Pfam (Ver 33.1) search. The Motifs identified by MEME were mapped against the query sequences using MAST(Ver. 5.4.1) to obtain the coordinates of motifs in each gad sequence [15].

### Logo plot & T cell receptor epitopes

The multiple sequence alignment obtained was converted into a logo plot using Weblogo (Ver 2.8.2) [18]. Here, each logo consists of a stack of symbols, and each symbol’s height is indicative of the amino acid conservation at a particular location. All motifs and T cell receptor epitope coordinates, obtained in the previous steps, were marked on the logo plot. The plot was then trimmed, with a special emphasis on regions with high overlap between motifs and T cell receptor epitopes.

## Results

### Multiple sequence alignment highlights several conserved residues between human and bacterial GAD

**Fig 1:**
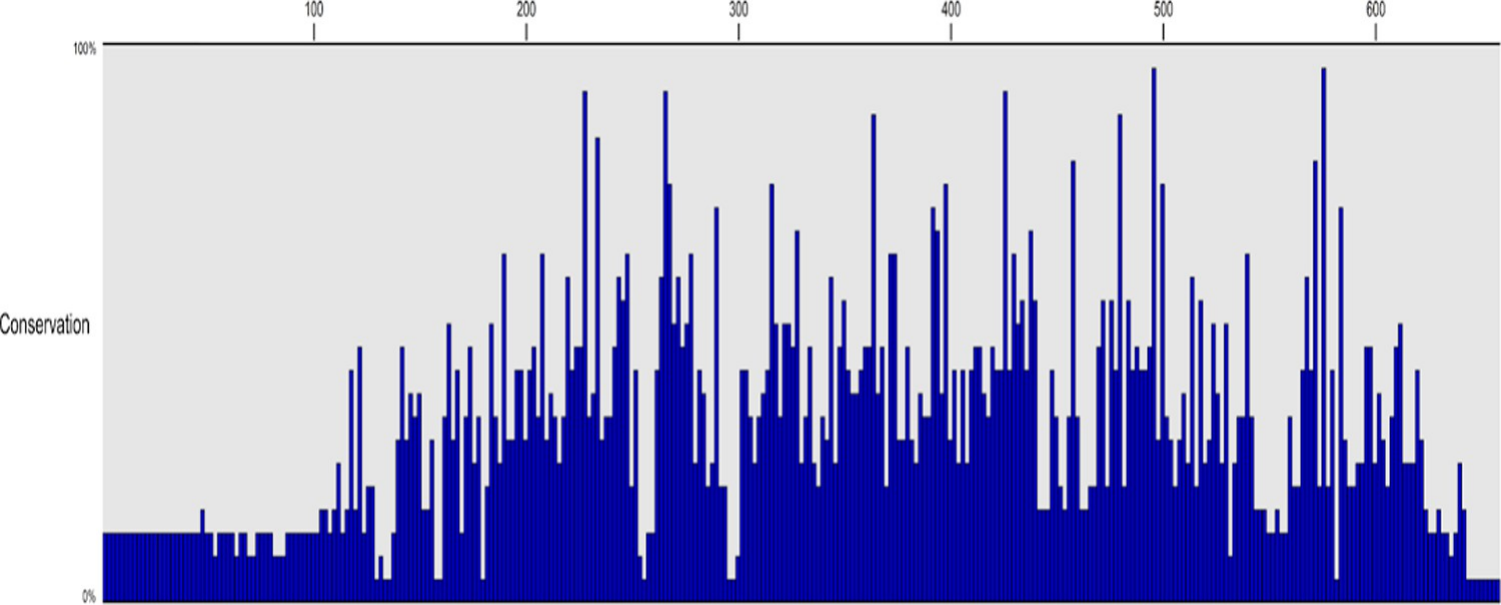
Multiple sequence alignment of twenty bacterial GAD proteins and three animal GAD65 proteins highlights conserved residues. Using a conservation histogram, across a diverse number of bacterial species and 3 animal species, 12 amino acids were found to be 100% conserved. Amino acids 1–110 are likely signal peptides required for eukaryotic protein translation and cellular transport not needed in bacteria. H275 and K276 are key residues required for PDD binding and are conserved in human GAD65 and bacterial GAD.

A total of 25 GAD protein sequences, including three mammalian GAD65 (human, chimpanzee, and mouse) and 22 bacterial GAD, were aligned (Table 1 & Fig 1). The three mammalian GAD65 sequences exhibited high conservation, while 20 out of the 22 bacteria GAD sequences exhibited high similarity to one another (for detailed conservation, see S1 Fig). Two bacteria, Bdellobibrio sp. Qaytius and Sediminibacterium spp., did not exhibit any protein conservation, which may be due to a misannotation within the RefSeq genome. We also found interesting similarities and differences between the mammalian and bacterial GAD sequences. There were two regions of interest: AA389-397 and AA418-425. AA389-397 contained several charged residues interspaced with hydrophobic residues, potentially a conserved alpha-helix structure. AA418-AA425 contains conserved HK residues. These two residues represent a known pyridoxal phosphate-binding site in E.coli GAD (H275 and K276) that is likely conserved in mammalian GAD65. Mammalian GAD65 was ~120 amino acids longer than bacterial GAD. Based on our alignment, this difference is mainly centered around the N-terminus, which suggests adaptations and inclusions of signal peptides required for complex cellular trafficking in mammalian cells. Also, we identified a few small gaps within the core regions of GAD between species, and these gaps are unlikely of any biological significance.

### Motif analysis uncovers regional similarities of human and bacterial GAD enzymatic regions

**Fig 2:**
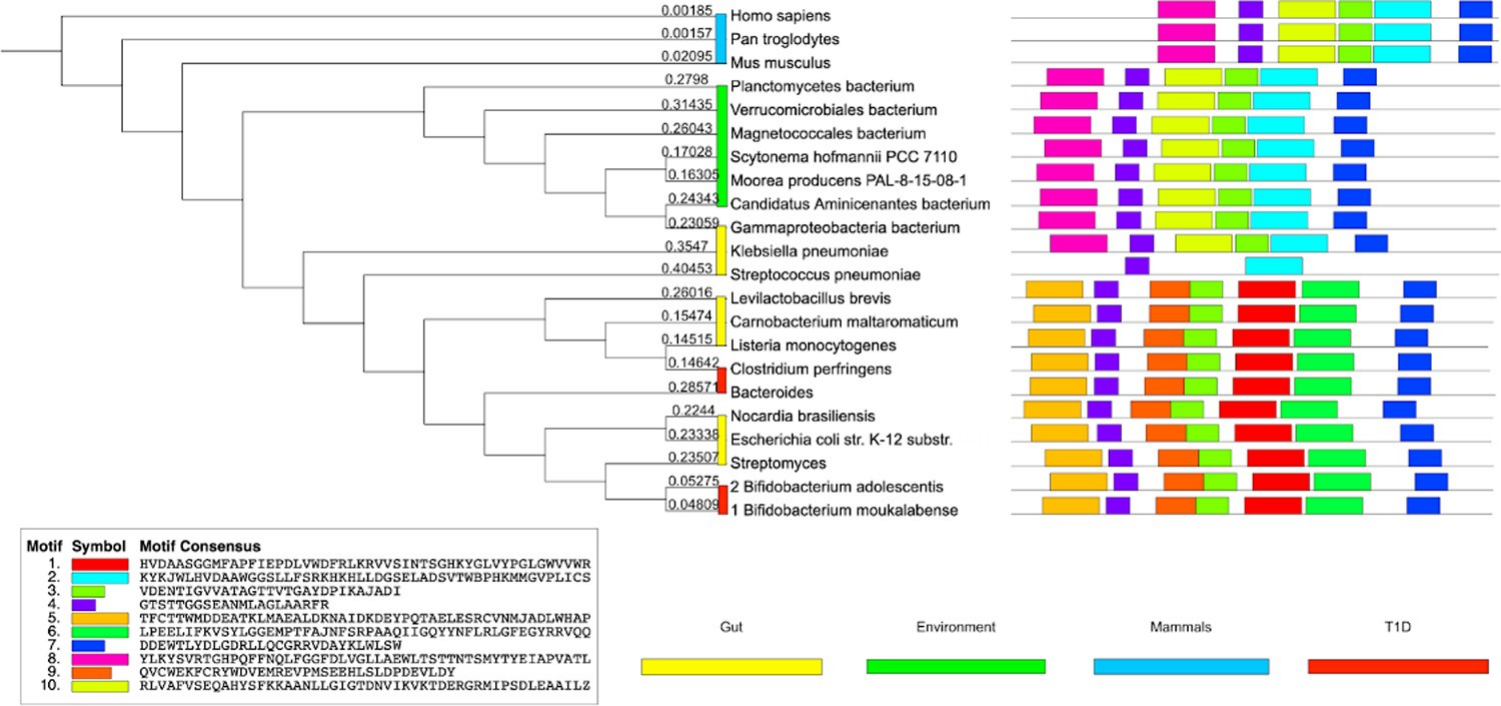
Motif discovery analysis of 19 bacterial GAD proteins and 3 animal GAD65 proteins to identify motifs conserved across various species and their functional relevance with GAD protein. Across all 22 GAD sequences under consideration, 10 motifs were each found in at least 10 of the sequences used for motif discovery. Each motif appears in a separate color below with the individual sequences described as well. Furthermore, the GAD sequences have been grouped based on the organism category from Table 1, and the respective categories have been represented as colored bars next to the organism name. In addition, 2 of the 10 motifs were found in all of the sequences. Each motif was then queried for using Pfam and CDD searches, and it was found that all 10 of the motifs hit the PDD domain in the GAD65 protein. All motifs match the pyridoxal-dependent decarboxylase (PDD) conserved domain.

Our motif analysis found 10 ungapped motifs, with each motif present in at least 10 of the sequences under consideration (Fig 2). Even though multiple common motifs between human GAD65 and bacterial GAD can be observed, it is noteworthy that 2 motif blocks (motifs 4 and 7) are conserved all across the organisms considered, which is indicative of the sequence similarity observed in the multiple sequence alignment. In addition, except for Streptococcus pneumoniae, motif 3 was consistently present in all sequences. The motifs were also grouped based on the phylogenetic tree constructed from the Multiple Sequence Alignment of GAD sequences. Six motif blocks (motifs 2, 3, 4, 7, 8, and 10) were conserved across three mammalian species, 6 environmental bacterial species, and 2 gut bacterial species. In addition, with exception of Streptococcus pneumoniae, 7 motif blocks were conserved across 4 T1D related bacterial species and 6 gut bacterial species. Furthermore, these motifs were queried on the Pfam and the CDD databases. All 10 motifs were identified as matches to the pyridoxal-dependent decarboxylase (PDD) conserved domain, which is instrumental in GABA biosynthesis from glutamate. Therefore, we show that human GAD65 and bacterial GAD share sequence similarities in a functional region of GAD.

### Overlap exists between T cell receptor epitopes of GAD65 and bacterial GAD

**Fig 3:**
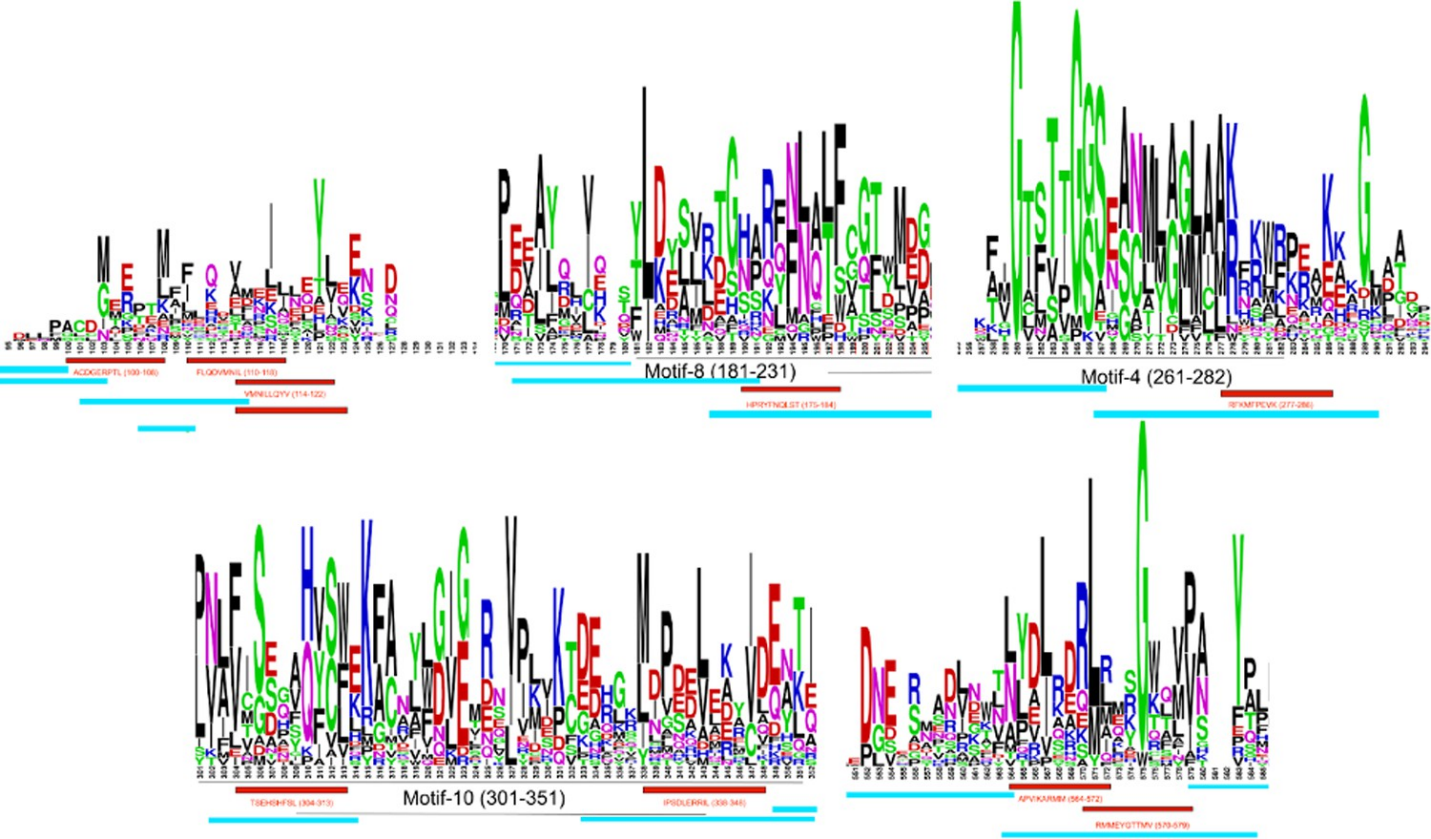
Conserved regions of GAD share common CD8+ and CD4+ T cell receptor epitopes. A total of 5 regions of interest were identified by overlapping T cell receptor epitopes to the sequence logo of 3 representative bacterial GAD and 3 animal GAD65 proteins. Three regions contained a motif match to Group II pyridoxal-dependent decarboxylases. The red and blue bars correspond to CD8+ and CD4+ T cell receptor epitopes, respectively. The black bars correspond to motif blocks with the motif numbers present in Fig 2. The first and last regions did not have any functional motifs, but they overlap with receptor epitopes. Of the amino acids in which both CD4+ and CD8+ receptor epitopes overlap, at least 1 amino acid residue is conserved across bacterial GAD and GAD65. Red is CD8+ epitopes, and Blue is CD4+ epitopes.

**Fig 4:**
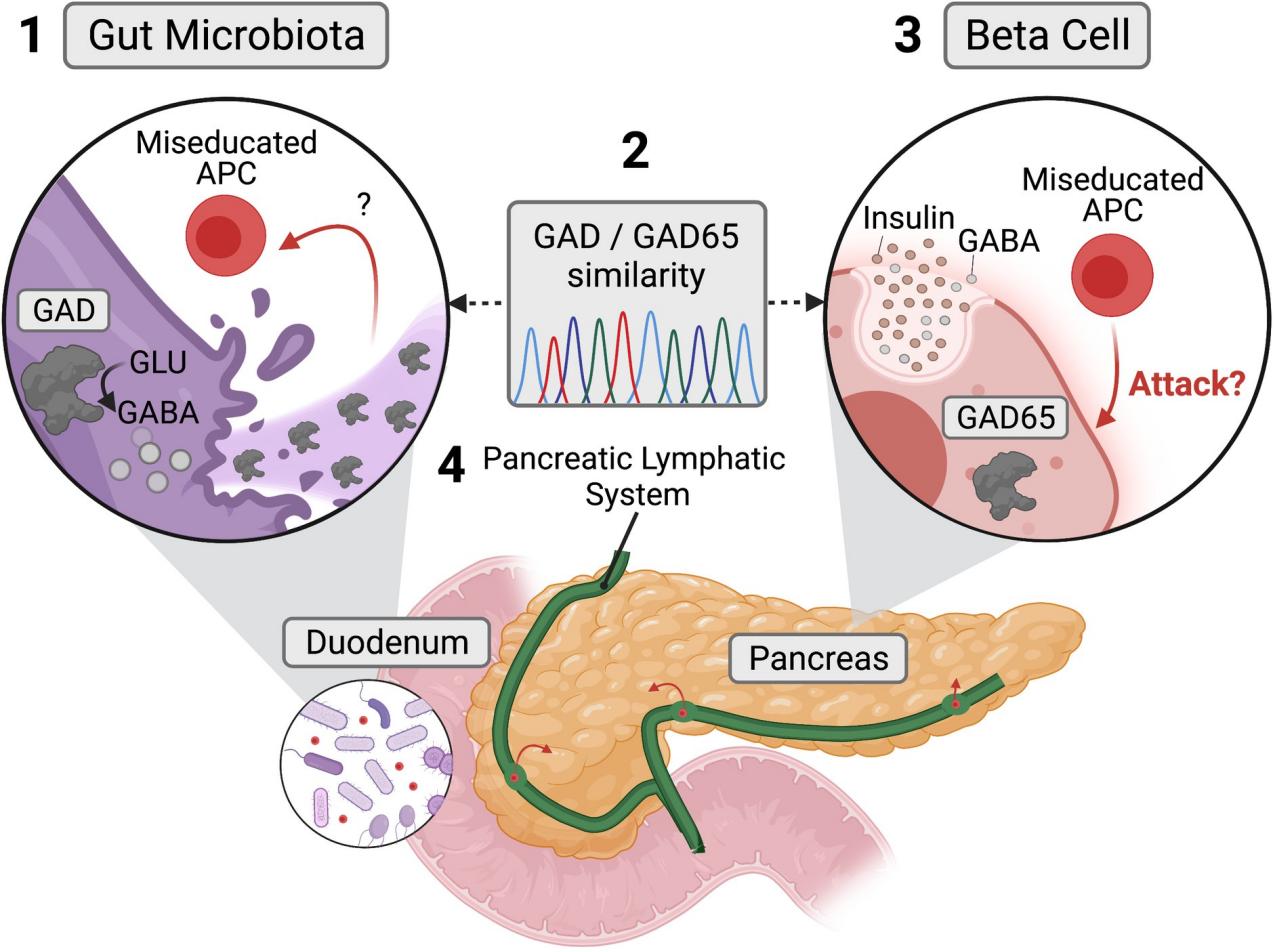
Miseducation of antigen-presenting cells (APCs) by bacterial GAD may contribute to pancreatic beta cell immune-mediated destruction. (1) The death of GABA-producing bacteria at the onset of T1D may release bacterial GAD leading to the miseducation of APCs due to the similarity between bacterial GAD and human GAD65 (2). Therefore, this miseducation could provoke the immune system to attack and produce antibodies targeting human GAD65 expressing beta cells (3). The pancreatic lymphatic system could provide a pathway for these miseducated APCs to traverse from the duodenum into pancreatic islets (4).

To compare the overlap between T cell receptor epitopes, human GAD65, and bacterial GAD, a logo plot of the multiple sequence alignment was made using three representative bacterial GAD and three animal GAD65 proteins. Motif positions for each sequence were manually annotated using the motif coordinates obtained in motif discovery and enrichment analysis. Thereafter, the logo plot was annotated with T cell receptor epitopes, and the overlapping regions were observed. The fine lines indicate the motif coordinates, while the red blocks indicate the CD8+ epitopes and blue blocks indicate the CD4+ epitopes. Three regions overlapped with a motif, which hit the PDD domain as stated in the motif analysis result description (Fig 3), confirmed by a CDD search. Of the amino acids in which both CD4 and CD8 receptor epitopes overlap, at least one amino acid residue is conserved across bacterial GAD and GAD65 (Fig 4). Our overlap findings between bacterial GAD and human GAD65 emphasize a possible mechanism for how T cells could recognize both bacterial and human GAD65.

## Discussion

This study explored the sequence similarities between GAD in twenty GABA-producing bacteria and human GAD65. We found that human and bacterial GAD have similar motifs and conserved residues. We identified notable conservation centered around the pyridoxal-dependent decarboxylase (PDD) domain of GAD65 found in pancreatic beta cells, particularly around two key substrate-binding residues. Moreover, some conserved sequences in bacterial GAD overlap with known human GAD65 T cell receptor epitopes. For example, GAD65-specific HLA-DR4 (DRB1*0401)–restricted murine T cell hybridoma line T33.1 recognizes the GAD65 274–286 epitope, and this epitope was conserved across bacteria and human samples examined here. Our *in silico* proof-of-concept suggests a possible relationship between the disappearance of GAD containing GABA-producing bacteria in the human gut microbiome and the appearance of GAD65 autoantibodies during the onset of T1D that needs to be further elucidated.

Our study investigates the genomic and residue similarities in the GAD gene of 29 bacteria, 12 of which have changed abundances in the gut microbiome of those with T1D (Table 1). Phylogenetically, the GAD65 sequences are separated into three distinct groups: eukaryotic GAD65, environmental bacterial GAD, and human-related bacterial GAD. Interestingly, we found that environmental bacterial GAD is closer to humans phylogenetically than gut and T1D-associated bacterial GAD. The “hygiene” hypothesis postulates that exposure to environmental bacteria and/or infectious diseases may ward off autoimmune diseases like T1D, highlighting another potential mechanism for immune recognition of GAD [19]. Nevertheless, gut and T1D bacterial species shared 3 motif blocks with mammalian GAD, suggesting an appreciable amount of sequence and motif conservation.

Additionally, we found that a residue within the GAD65 catalytic loop, Lys396, aligned across both bacteria GAD and human GAD65. Lys396 covalently binds PDP, a GAD/GAD65 co-factor essential for GABA production. When GAD65 is devoid of the PLP co-factor, it shows higher antigenicity than GAD65 bound by PDP [20]. Therefore, PLP reduction through decreased levels of its precursor, vitamin B6, may provoke beta cell destruction mediated by miseducated GAD-activated T cells. Furthermore, beta cell-derived GABA may provide inhibitory signals for autoimmunity such that reduced GAD65 activity may cause an exacerbation of T1D immune pathology against beta cells [21].

The elimination of GABA-producing bacteria and emergence of GAD65 autoantibodies in T1D pathogenesis is explored here through *in silico* analyses. Healthy individuals have a greater gut bacterial diversity with GABA-producing pathways activated in several bacterial species compared to T1D donor stool samples [22]. Previous studies show that Bifidobacterium species are especially vulnerable to antibiotics, leading to investigations of how antibiotic treatments influence this major GABA producer [23]. The TEDDY study cohort of children with T1D found a decrease in the abundance of multiple Bifidobacterium bacterial species in participants following treatments with antibiotics [24]. Therefore, our findings of sequence and epitope similarities further support our paradigm that eliminating GAD-producing bacterial species in humans could correlate with the emergence of GAD65 autoimmunity and subsequent development of T1D.

Thus, we propose that bacterial GAD found in GABA-producing bacteria may act as an antigen to activate submucosal T cells due to gut microbiome bacterial destruction, either through viral- or antibiotic-mediated mechanisms [25]. Further, we posit that primed bacterial GAD T cells bind GAD65 in the islet environment due to the GAD and GAD65 epitope similarity. That leaves us to explore how GAD65, an intracellular enzyme found in beta cells, might get recognized by bacterial-derived anti-GAD T cells. Pre-clinical studies demonstrated that GAD65 could associate with the plasma membrane and enter the extracellular space [26, 27]. In this scenario, “deputized” beta cells that present GAD65 might have an opportunity to interact with GAD sensitized T cells [28, 29]. Therefore, one can imagine a scenario in which GAD primed T cells from the gut become initially alerted to the islet environment as GAD65 is presented via these deputized beta cells. Once alerted, GAD primed T cells could destroy beta cells that display GAD65 at the plasma membrane leading to the development of T1D.

It is important to note a few limitations within our analyses. Many gut microbial genomes and genome annotations were incomplete, particularly for some GABA-producing bacterial species of importance significantly changed in T1D. We did not include several species due to missing GAD genome annotations. Together, only 20 bacterial sequences were analyzed for their homology, with many environmental organisms. A larger cohort would further strengthen our sequence analyses and hypotheses. Furthermore, we hypothesized that the change in anti-GAD65 is partly due to the changes in the gut microbiome. Additional studies are needed to determine what insult(s) would cause such a disturbance and what cellular mechanisms are involved in the miseducation of immune cells due to the release of GAD in the gut. The window for the disappearance of the GAD-containing bacterial species needs to be identified to determine if it precedes or follows the appearance of autoantibodies to GAD65 in humans. Lastly, the analysis relied on known and curated T cell epitopes of GAD65 from a literature review, in which we demonstrated similarity to bacterial GAD. Whether or not the bacterial peptides bind to human T cell receptors must be further verified *in vitro*.

Taken together, our in silico analyses of genome sequences and motifs from bacterial, human, and T cell epitopes identified several homologies that may inform further experimentation and inquiry into the relationship between T1D pathogenesis and microbiome dysbiosis.

## Supporting information

S1 Fig Multiple sequence alignment of twenty bacterial GAD proteins and three animal GAD65 proteins highlights conserved residues. Across a diverse number of bacterial species and 3 animal species, 12 amino acids were found to be 100% conserved. Amino acids 1–110 are likely signal peptides required for eukaryotic protein translation and cellular transport not needed in bacteria. H275 and K276 are key residues required for PDD binding and are conserved in human GAD65 and bacterial GAD.
(TIF)

S1 File (ZIP)

## References

[1] Petersen JS, Hejnaes KR, Moody A, et al. Detection of GAD65 antibodies in diabetes and other autoimmune diseases using a simple radioligand assay. Diabetes. 1994;43(3):459–467. doi: 10.2337/diab.43.3.459 8314020

[2] Fenalti G, Law RH, Buckle AM, et al. GABA production by glutamic acid decarboxylase is regulated by a dynamic catalytic loop. Nat Struct Mol Biol. 2007;14(4):280–286. doi: 10.1038/nsmb1228 17384644

[3] Rubí B. (2012). Pyridoxal 5’-phosphate (PLP) deficiency might contribute to the onset of type I diabetes. Medical hypotheses, 78(1), 179–182. doi: 10.1016/j.mehy.2011.10.021 22088923

[4] Sommer F, Bäckhed F. The gut microbiota—masters of host development and physiology. Nat Rev Microbiol. 2013;11(4):227–238. doi: 10.1038/nrmicro2974 23435359

[5] Duranti S, Ruiz L, Lugli GA, et al. Bifidobacterium adolescentis as a key member of the human gut microbiota in the production of GABA. Sci Rep. 2020;10(1):14112. doi: 10.1038/s41598-020-70986-z 32839473 PMC7445748

[6] Zhou H, Sun L, Zhang S, Zhao X, Gang X, Wang G. Evaluating the Causal Role of Gut Microbiota in Type 1 Diabetes and Its Possible Pathogenic Mechanisms. Front Endocrinol (Lausanne). 2020;11:125. doi: 10.3389/fendo.2020.00125 32265832 PMC7105744

[7] de Goffau MC, Fuentes S, van den Bogert B, et al. Aberrant gut microbiota composition at the onset of type 1 diabetes in young children. Diabetologia. 2014;57(8):1569–1577. doi: 10.1007/s00125-014-3274-0 24930037

[8] Murri M, Leiva I, Gomez-Zumaquero JM, et al. Gut microbiota in children with type 1 diabetes differs from that in healthy children: a case-control study. BMC Med. 2013;11:46. doi: 10.1186/1741-7015-11-46 23433344 PMC3621820

[9] Leiva-Gea I, Sánchez-Alcoholado L, Martín-Tejedor B, et al. Gut Microbiota Differs in Composition and Functionality Between Children With Type 1 Diabetes and MODY2 and Healthy Control Subjects: A Case-Control Study. Diabetes Care. 2018;41(11):2385–2395. doi: 10.2337/dc18-0253 30224347

[10] Prause M, Pedersen SS, Tsonkova V, Qiao M, Billestrup N. Butyrate Protects Pancreatic Beta Cells from Cytokine-Induced Dysfunction. Int J Mol Sci. 2021;22(19):10427. doi: 10.3390/ijms221910427 34638768 PMC8508700

[11] Harbison JE, Roth-Schulze AJ, Giles LC, et al. Gut microbiome dysbiosis and increased intestinal permeability in children with islet autoimmunity and type 1 diabetes: A prospective cohort study. Pediatr Diabetes. 2019;20(5):574–583. doi: 10.1111/pedi.12865 31081243

[12] Soyucen E, Gulcan A, Aktuglu-Zeybek AC, Onal H, Kiykim E, Aydin A. Differences in the gut microbiota of healthy children and those with type 1 diabetes. Pediatr Int. 2014;56(3):336–343. doi: 10.1111/ped.12243 24475780

[13] James Eddie A., Mallone Roberto, Kent Sally C., Teresa P. DiLorenzo; T-Cell Epitopes and Neo-epitopes in Type 1 Diabetes: A Comprehensive Update and Reappraisal. Diabetes 1 July 2020; 69 (7): 1311–1335. doi: 10.2337/dbi19-0022 32561620 PMC7306130

[14] Thompson J D et al. “CLUSTAL W: improving the sensitivity of progressive multiple sequence alignment through sequence weighting, position-specific gap penalties and weight matrix choice.” Nucleic acids research vol. 22,22 (1994): 4673–80. doi: 10.1093/nar/22.22.4673 7984417 PMC308517

[15] Bailey Timothy L., Johnson James, Grant Charles E., Noble William S., "The MEME Suite", Nucleic Acids Research, 43(W1):W39–W49, 2015. doi: 10.1093/nar/gkv416 25953851 PMC4489269

[16] EMBL-EBI. Simple phylogeny. EBI. https://www.ebi.ac.uk/Tools/phylogeny/simple_phylogeny/

[17] iTOL V6. https://itol.embl.de/

[18] Crooks, Gavin E et al. “WebLogo: a sequence logo generator.” Genome research vol. 14,6 (2004): 1188–90. doi: 10.1101/gr.849004 15173120 PMC419797

[19] Mishra SP, Wang S, Nagpal R, et al. Probiotics and Prebiotics for the Amelioration of Type 1 Diabetes: Present and Future Perspectives. Microorganisms. 2019;7(3):67. doi: 10.3390/microorganisms7030067 30832381 PMC6463158

[20] Towns R, Pietropaolo M. GAD65 autoantibodies and its role as biomarker of Type 1 diabetes and Latent Autoimmune Diabetes in Adults (LADA). Drugs Future. 2011;36(11):847. doi: 10.1358/dof.2011.036.11.1710754 22869930 PMC3411186

[21] Bhat R, Axtell R, Mitra A, et al. Inhibitory role for GABA in autoimmune inflammation. Proc Natl Acad Sci U S A. 2010;107(6):2580–2585. doi: 10.1073/pnas.0915139107 20133656 PMC2823917

[22] Strandwitz P, Kim KH, Terekhova D, et al. GABA-modulating bacteria of the human gut microbiota. Nat Microbiol. 2019;4(3):396–403. doi: 10.1038/s41564-018-0307-3 30531975 PMC6384127

[23] Pokusaeva K, Johnson C, Luk B, et al. GABA-producing Bifidobacterium dentium modulates visceral sensitivity in the intestine. Neurogastroenterol Motil. 2017;29(1):e12904. doi: 10.1111/nmo.12904 27458085 PMC5195897

[24] Vatanen T, Franzosa EA, Schwager R, et al. The human gut microbiome in early-onset type 1 diabetes from the TEDDY study. Nature. 2018;562(7728):589–594. doi: 10.1038/s41586-018-0620-2 30356183 PMC6296767

[25] Pereira MS, Redanz S, Kriegel MA. Skin Deep: The Role of the Microbiota in Cutaneous Autoimmunity. J Invest Dermatol. 2022; S0022-202X(21)02612-9. doi: 10.1016/j.jid.2021.12.005 35027173

[26] Jin H, Wu H, Osterhaus G, et al. Demonstration of functional coupling between gamma -aminobutyric acid (GABA) synthesis and vesicular GABA transport into synaptic vesicles. Proc Natl Acad Sci U S A. 2003;100(7):4293–4298. doi: 10.1073/pnas.0730698100 12634427 PMC153086

[27] Christgau S, Aanstoot HJ, Schierbeck H, et al. Membrane anchoring of the autoantigen GAD65 to microvesicles in pancreatic beta-cells by palmitoylation in the NH2-terminal domain. J Cell Biol. 1992;118(2):309–320. doi: 10.1083/jcb.118.2.309 1321158 PMC2290048

[28] Li Y, Sun F, Yue TT, et al. Revisiting the Antigen-Presenting Function of β Cells in T1D Pathogenesis. Front Immunol. 2021;12:690783. doi: 10.3389/fimmu.2021.690783 34335595 PMC8318689

[29] Cianciaruso C, Phelps EA, Pasquier M, et al. Primary Human and Rat β-Cells Release the Intracellular Autoantigens GAD65, IA-2, and Proinsulin in Exosomes Together With Cytokine-Induced Enhancers of Immunity. Diabetes. 2017;66(2):460–473. doi: 10.2337/db16-0671 27872147

