# Supplementary figures and images for: Similarities between bacterial GAD and human GAD65: Implications in gut mediated autoimmune type 1 diabetes
Source: PLoS One. 2022 Feb 23;17(2):e0261103. doi: 10.1371/journal.pone.0261103 (PMC8865633; doi:10.1371/journal.pone.0261103)

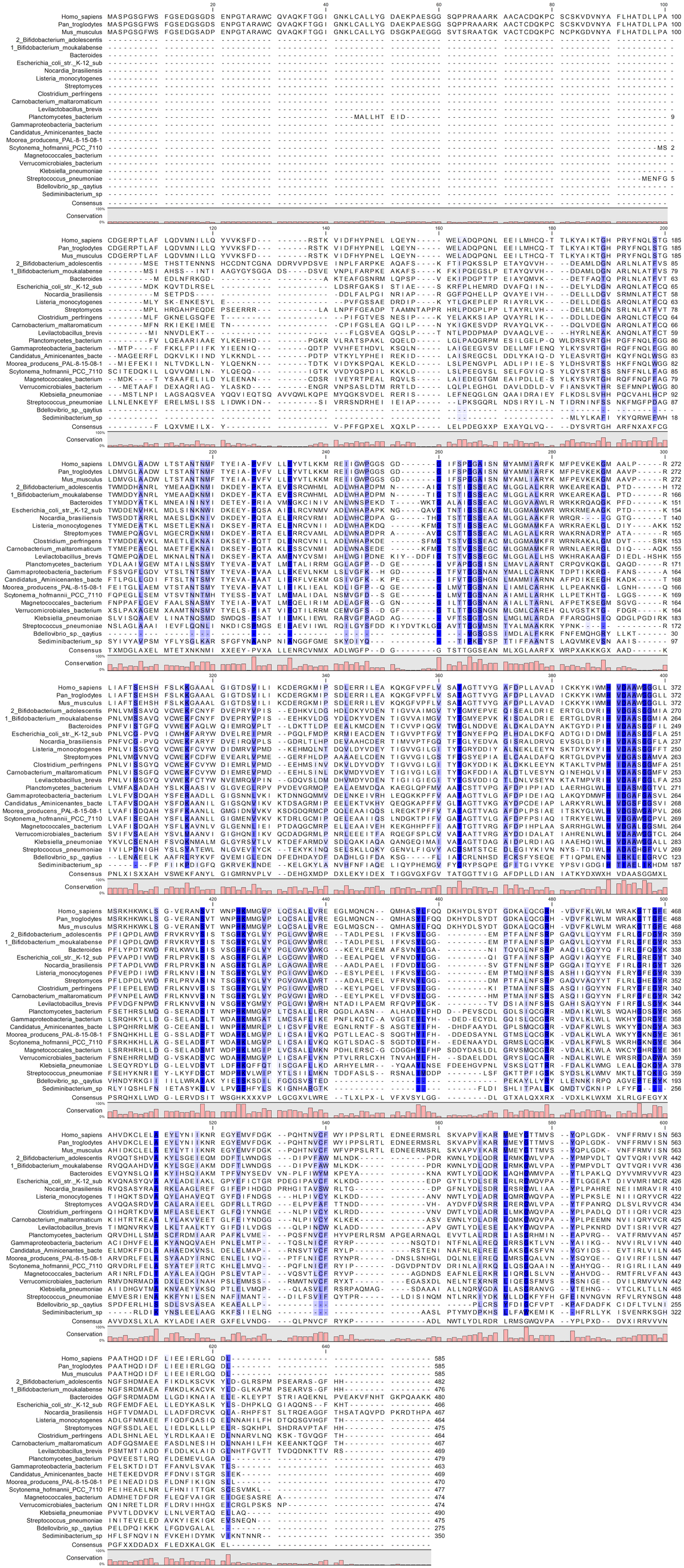

Supplement: S1 Fig — Across a diverse number of bacterial species and 3 animal species, 12 amino acids were found to be 100% conserved. Amino acids 1–110 are likely signal peptides required for eukaryotic protein translation and cellular transport not needed in bacteria. H275 and K276 are key residues required for PDD binding and are conserved in human GAD65 and bacterial GAD. (TIF) [file pone.0261103.s001.tif]
